# A microwave assisted method to synthesize nanoCoFe_2_O_4_@methyl cellulose as a novel metal-organic framework for antibiotic degradation

**DOI:** 10.1016/j.mex.2019.06.017

**Published:** 2019-06-26

**Authors:** Alireza Nasiri, Fatemeh Tamaddon, Mohammad Hossein Mosslemin, Maryam Faraji

**Affiliations:** a Environmental Health Engineering Research Center, Kerman University of Medical Sciences, Kerman, Iran; b Department of Chemistry, Islamic Azad University, Yazd Branch, Yazd, Iran; c Department of Chemistry, Faculty of Science, Yazd University, Yazd, 89195-741, Iran; d Department of Environmental Health, School of Public Health, Kerman University of Medical Sciences, Kerman, Iran

**Keywords:** Synthesis of nanoCoFe_2_O_4_@MC as a novel nanophotocatalyst by a microwave-assisted method for metronidazole photodegradation, CoFe_2_O_4_@methyl cellulose, Nanobiomagnetic photocatalyst, Microwave, Metronidazole, Water treatment, Photocatalytic degradation

## Abstract

In this research, magnetically separable nanoCoFe_2_O_4_@methyl cellulose (MC) as a novel metal-organic framework was designed by a facile, fast, and new microwave-assisted method and then characterized. To assay the photocatalytic activity of nanoCoFe_2_O_4_@MC, its ability in metronidazole (MNZ) removal was investigated by considering the effect of some variables such as initial MNZ concentrations (5–20 mg/L), pH (3–11), nanophotocatalyst loading (0.0–0.4 g), and reaction time (15–120 min). The kinetic performance of the process was assessed by the *pseudo-*first order and *Langmuir*-*Hinshelwood* models. The concentration of MNZ was determined by high performance liquid chromatography. The optimal conditions for the maximum MNZ removal efficiency (85.3%) included pH of 11, MNZ concentration of 5 mg/L, photocatalyst loading of 0.2 g, and irradiation time of 120 min. Moreover, the reusability and chemical stability of nanoCoFe_2_O_4_@MC were studied. MNZ was successfully degraded at a rate of 77.58% in the fourth run.

**Advantages of this technique were as follows:**

• A facile, fast, and new microwave-assisted method was developed to synthesize nanoCoFe_2_O_4_@MC as a new nanobiomagnetic photocatalyst.
• Pure-phase spinel ferrites, spherical particle morphology with smaller agglomeration, and ferromagnetic nature of nanoCoFe_2_O_4_@MC were confirmed.
• NanoCoFe_2_O_4_@MC displayed a significant photocatalytic activity in the photocatalytic degradation of MNZ; moreover, it was easily separated by a magnet and exhibited good chemical stability.

## Graphical abstract

Mechanism for photodegradation of MNZ by nanoCoFe_2_O_4_@MC.

**Figure:**
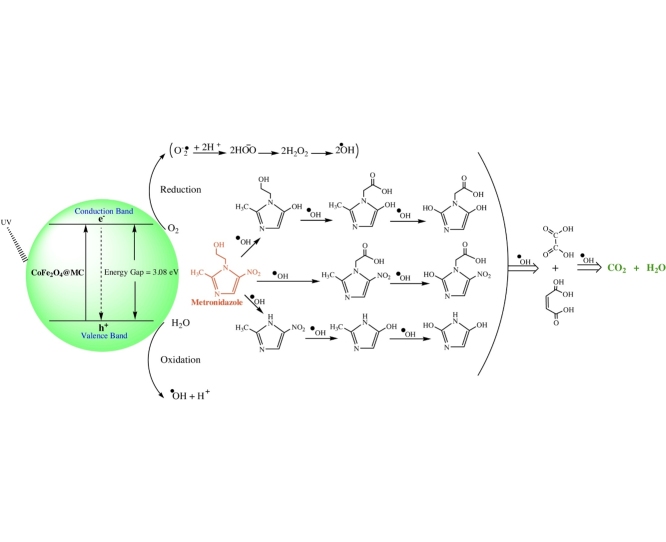

**Specifications Table**

**Table:** 

Subject Area:	*Environmental Sciences*
More specific subject area:	*Chemical engineering in environmental sciences*
Method name:	*Synthesis of nanoCoFe_2_O_4_@MC as a novel nanophotocatalyst by a microwave-assisted method for metronidazole photodegradation*
Name and reference of original method:	*- Nasiri A, Tamaddon F, Mosslemin MH, Gharaghani MA, Asadipour A. New magnetic nanobiocomposite CoFe_2_O_4_@methylcellulose: Facile synthesis, characterization, and photocatalytic degradation of metronidazole. Journal of Materials Science: Materials in Electronics. (2019); 30(9):8595–8610.* *- Nasiri A, Tamaddon F, Mosslemin M H, Amiri Gharaghani M, Asadipour A. Magnetic nanobiocomposite CuFe_2_O_4_@methylcellulose prepared as a new nano-photocatalyst for degradation of ciprofloxacin from an aqueous solution. Environmental Health Engineering and Management Journal. (2019); 6(1):41-51.*
Resource availability:	*NA*

## Method

**Fig. 1:**
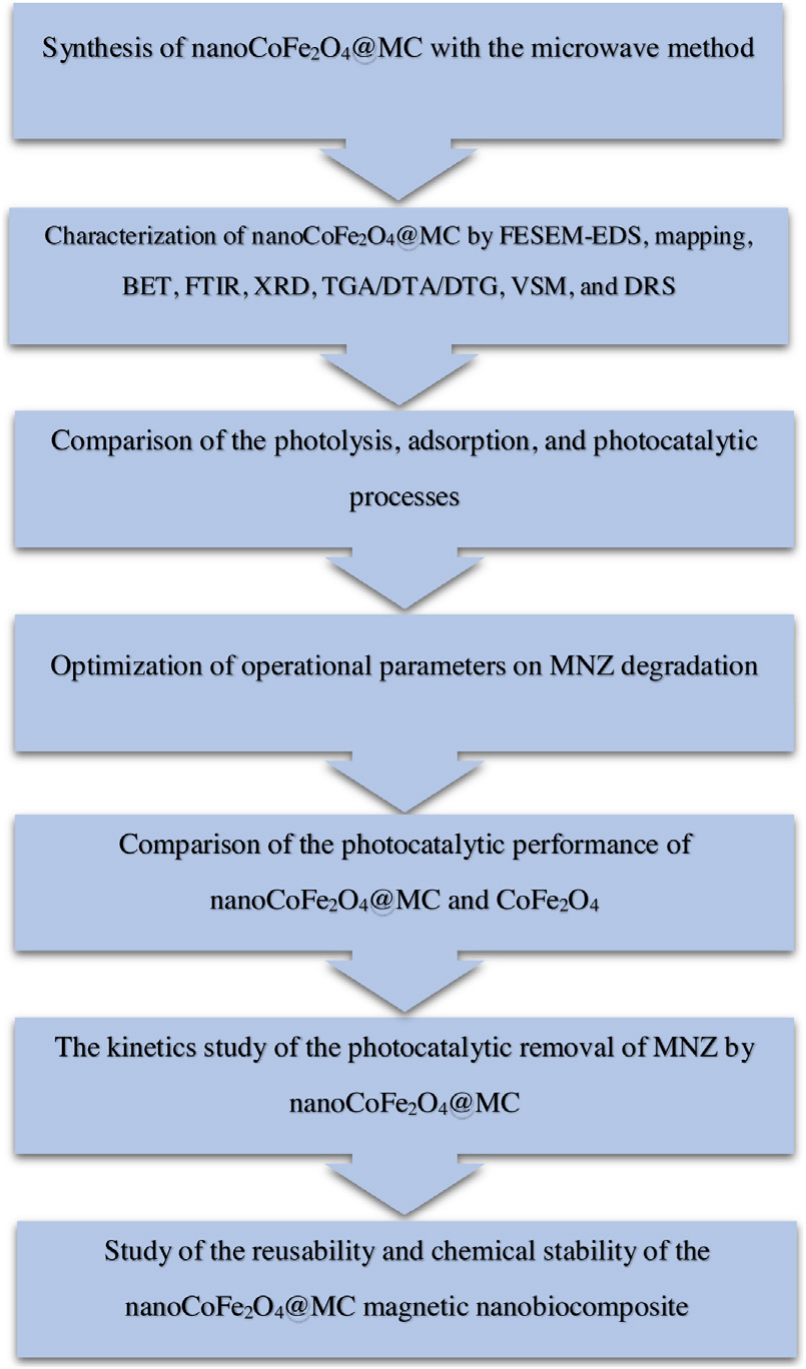
The flow diagram of the study stages.

Photocatalytic degradation processes are effective and widely-used methods for treatment of water and wastewater containing persistent compounds such as antibiotics [[1], [2], [3]]. There are few reports on photocatalytic degradation of metronidazole (MNZ) in water. Some of these reports were pH-sensitive and in some others, an extra chemical oxidant was used to increase degradation efficiency [[4], [5], [6], [7]]. Thorough review of the literature yielded no research regarding MNZ removal from aqueous solutions using metal-organic frameworks (MOFs). MOFs are a new class of hybrid materials built from organic linkers and inorganic metal nodes through coordination bonds [[8], [9], [10]]. Thus, to address these issues, a magnetically separable nanoCoFe_2_O_4_@methyl cellulose (MC) photocatalyst was designed by a facile, fast, and new microwave-assisted method. The study stages were as follows: synthesis and characterization of nanoCoFe_2_O_4_@MC; comparison of the photolysis, adsorption, and photocatalytic processes; study of the effects of operational parameters on the MNZ removal efficiency; comparison of the photocatalytic performance of nanoCoFe_2_O_4_@MC and CoFe_2_O_4_; study of kinetics of the photocatalytic removal of MNZ by nanoCoFe_2_O_4_@MC; and study of the recovery, reusability and chemical stability of nanoCoFe_2_O_4_@MC. The flow diagram of the study stages is exhibited in Fig. 1.

### Chemicals

MC, CoCl_2_·6H_2_O, NaOH, and FeCl_3_·6H_2_O were obtained from Merck Company (Germany) and used without further purification. MNZ with a purity of 99% was purchased from Tamad Pharmaceutical Company (Tehran, Iran). Other chemicals such as HCl, NaOH, acetonitrile, acetic acid, methanol, and ethanol were obtained from Merck Company (Germany). All the materials were in analytical grade and used without further purification. Deionized water was used to make solutions.

### Synthesis and characterization of nanoCoFe_2_O_4_@MC

**Fig. 2:**
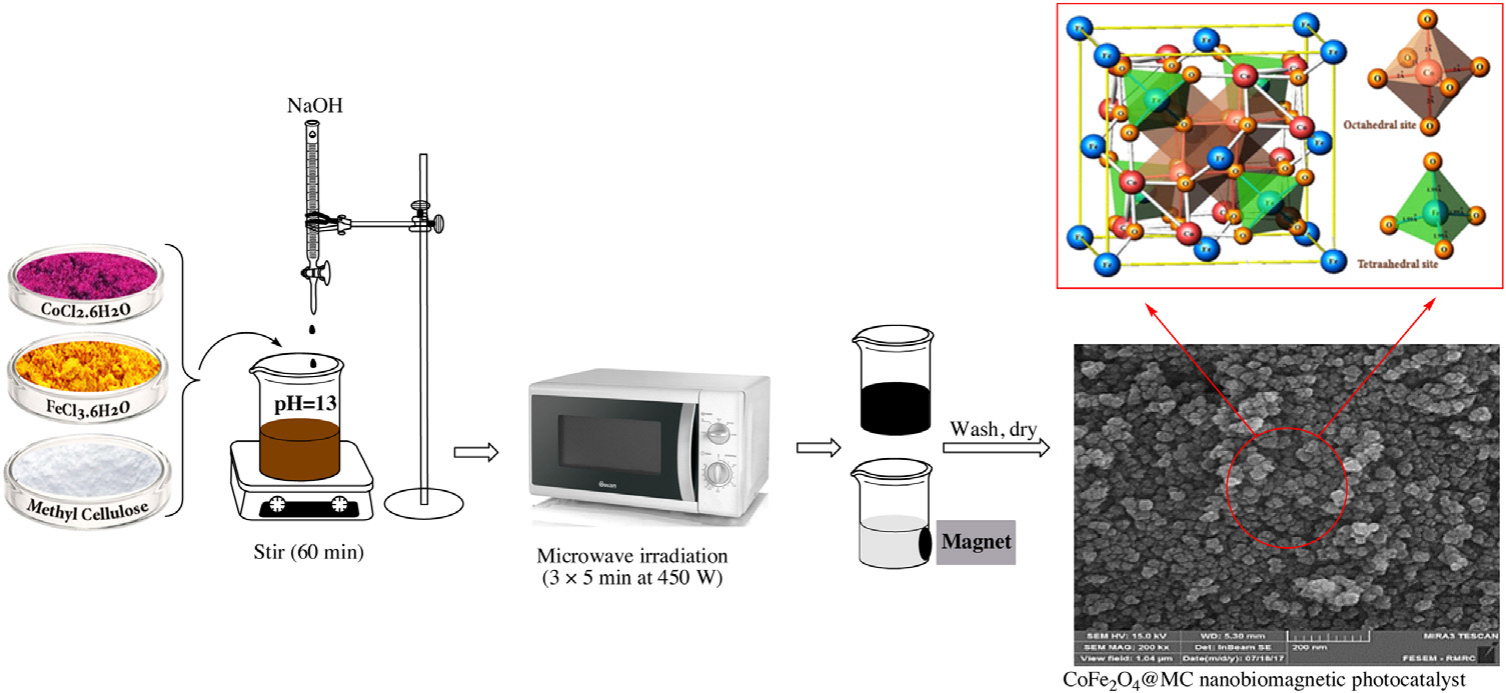
The schematic illustration of the synthesis of nanoCoFe_2_O_4_@MC as a new magnetic nanophotocatalyst.

FeCl_3_.6H_2_O and CoCl_2_.6H_2_O in a 2:1 ratio were dissolved in 50 mL deionized water. Then, MC was added to the solution, and the mixture was vigorously stirred at room temperature. After that, NaOH was added to the suspension within 1 h to adjust the pH at 13. Dark brown solutions were subjected to microwave irradiation (3 × 5 min at 450 W) (Samsung Microwave ME201, 20 L). Afterwards, nanoCoFe_2_O_4_@MC was precipitated as a lightweight massive powder. The obtained magnetic solids were separated by using an external magnet and then washed with deionized water many times. Afterwards, they were dried at 100 °C in a vacuum oven for 24 h [11] (Fig. 2). The Fourier transform infrared spectroscopy (FT-IR) of the samples was obtained using a FT-IR 6300 Brucker, and the X-ray powder diffraction (XRD) of nanoCoFe_2_O_4_@MC was recorded in the diffraction angle range of 2*θ* = 10^○^–80^○^ by an X'Pert PRO MPD PAnalytical using Ni-filtered Cu Kα radiation. Thermal gravimetric analysis (TGA), derivative thermogravimetric analysis (DTG), and differential thermal analysis (DTA) were carried out using an STA (PC Luxx 409-NETZSCH) instrument at the rate of 10 °C min^−1^ in air. The magnetic properties of nanoCoFe_2_O_4_@MC were characterized by a vibrating sample magnetometer (VSM) (LakeShore Cryotronics-7404) at room temperature. The microstructure, morphology, and chemical composition of nanoCoFe_2_O_4_@MC were investigated by field emission scanning electron microscope-energy dispersive spectroscopy (FESEM-EDS) (MIRA3TESCANXMU). The EDS mapping was employed to further confirm the components and element distribution (MIRA3TESCANXMU). Moreover, the Brunauer-Emmett-Teller (BET) surface areas were evaluated based on N_2_ adsorption-desorption isotherms using a specific surface analyzer (BELSORP-mini II) at 120 °C. The UV–vis diffuse reflectance spectra (UV-DRS) of the samples were taken by a UV–vis spectrophotometer (Shimadzu, UV-2550). The quantity of the dissolved Fe and Co ions in leachate was determined with a flame atomic absorption spectrometer (PG Instruments, model PG 990, England) at the wavelength of 240.7 nm and 248.3 nm, respectively. A high performance liquid chromatography (HPLC) device (YL 9100 Waters, USA) was utilized to identify and measure MNZ [11]. Standard MNZ with a purity of 99% was used to adjust and run the device. In addition, acetonitrile and deionized water were used as mobile phases with volume ratios of 30:70. The utilized column was C_18_ with particles of 5 μm size, length of 250 mm, and internal diameter of 4.6 mm. MNZ was identified by a UV absorbance detector within the wavelength of 348 nm with an injection volume of 20 μL and flow rate of 1 mL/min (Table 1).

**Table 1:** Details of the HPLC analysis.

Characteristic	Condition
Detector	UV absorbance at the wavelength of 348 nm
Column model	C_18_ column with 5 μm particles
Column characteristic	250 mm length and 4.6 mm internal diameter
Mobile phase	Acetonitrile : Deionized water (30:70, V/V)
Flow rate of mobile phase	1 mL/min
Volume of injection	20 μL

### Comparison of the photolysis, adsorption, and photocatalytic processes

In studies on photocatalysis processes, comparison of the results of the photolysis, adsorption and photocatalytic mechanisms is important [12,13]. Thus, the mentioned processes were respectively differentiated in the experiments in the presence of UV radiation, but without a catalyst to assay the photolysis process and in dark condition to evaluate the adsorption process. Based on the 23.7%, 16.9%, and 81.59% removal efficiency for adsorption, photolysis, and photocatalysis, respectively, MNZ removal by nanoCoFe_2_O_4_@MC occurs mainly through photocatalytic degradation.

### Optimization of operational parameters on the MNZ removal efficiency

**Fig. 3:**
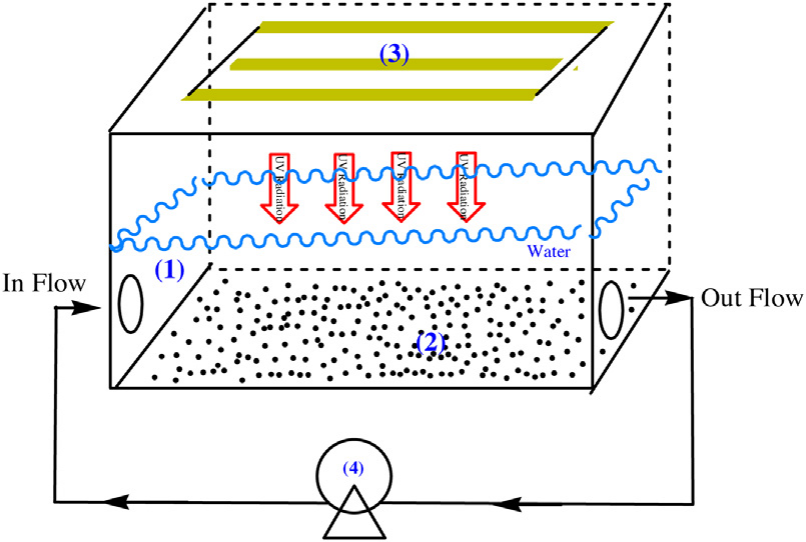
The photoreactor designed for the photocatalytic degradation of MNZ (1. The Plexiglas reactor, 2. the photocatalyst, 3. the UV-C lamp, and 4. the peristaltic pump).

The effects of initial MNZ concentrations (5, 10, 15, 20 mg/L), pH (3, 5, 7, 11), nanocatalyst loading (0.0, 0.1, 0.2, 0.3, 0.4 g), and UV-C irradiation time (15, 30, 45, 60, 75, 90, 105, 120 min) were optimized in a batch photoreactor. The photoreactor was a rectangular cubic shape, made from Plexiglas, and had internal dimensions of 25 cm (length), 10 cm (width), and 5 cm (height), an applicable volume of 300 mL, and three UV lamps (low pressure, 6 W, Philips) that were placed on top of the reactor. The reactor was designed so as to provide the minimum distance between the catalyst and the light supplier to generate more hydroxyl radicals through the excitation of the catalyst. A peristaltic pump with a flow of 1 mL/s was used to mix the reactor contents. The photoreactor designed for the current study is shown in Fig. 3. The samples were taken at the deﬁnite interval of times during the irradiation; after the separation of nanoCoFe_2_O_4_@MC by an external magnet, the samples were analyzed by HPLC. Then, the degradation efficiency (*η*) was calculated by Eq. (1):

(1) *η*% = 100 (C_0_ − C_t_)/C_0_

where C_t_ and C_0_ show the attained absorbance value of the MNZ solution at different periods of time (t) and at zero min, respectively, as measured by HPLC.

### Comparison of the photocatalytic performance of nanoCoFe_2_O_4_@MC and CoFe_2_O_4_

The photocatalytic performance of nanoCoFe_2_O_4_@MC and CoFe_2_O_4_ for degradation of MNZ was compared in the optimal conditions: pH of 11, irradiation time of 120 min, MNZ concentration of 5 mg/L, and photocatalyst loading of 0.2 g. The MNZ removal efficiency with the nanoCoFe_2_O_4_@MC and CoFe_2_O_4_ photocatalysts was 85.30% and 55.13%, respectively.

### Study of the kinetics of MNZ removal by nanoCoFe_2_O_4_@MC

The kinetics of MNZ removal by nanoCoFe_2_O_4_@MC was studied by the *pseudo-*first order (Eq. (2)) and *Langmuir*-*Hinshelwood* (Eq. (3)) kinetic models, as described in Table 2 [14,15]. The kinetic linear models showed that nanoCoFe_2_O_4_@MC catalyzed MNZ removal, following either the *pseudo*-first order kinetic model or the *Langmuir*-*Hinshelwood* equation. According to the *pseudo*-first order kinetic model, the correlation coefficient (R^2^) for concentrations of 5, 10, 15, 20, and 25 mg/L was 0.97, 0.99, 0.96, 0.85 and 0.91, respectively. The equilibrium adsorption coefficient of *Langmuir*-*Hinshelwood* and the rate constant of the superficial reaction were obtained as 0.594 L mg^−1^ and 0.015 mg L^−1^ min^−1^, respectively. The high correlation coefficient (R^2^ = 0.907) showed that the photocatalytic degradation of MNZ followed the *Langmuir*-*Hinshelwood* kinetic model.

**Table 2:** The *pseudo-*first order and Langmuir-Hinshelwood kinetic models.

Model	Formula	Parameters
*Pseudo-*first order	Ln (C_t_/C_0_) = −K_obs_t	C_0_ (mg/L): initial concentrations of MNZ
		C_t_ (mg/L): MNZ concentration at certain reaction times
		K_obs_ (min^−1^): constant rate of the *pseudo-*first order reaction
		t (min): reaction time

*Langmuir-Hinshelwood*	$\frac{1}{K_{\text{o}\text{b}\text{s}\;}}=\frac{1}{K_{c}K_{L-H}}+\frac{C_{0}}{K_{c}}$	K_c_ (mg/L min): constant rate of the superficial reaction
		K_L-H_ (L/mg): adsorption equilibrium constant of the L-H model

### Study of the reusability and chemical stability of nanoCoFe_2_O_4_@MC

Due to the importance of reusability and stability of the photocatalyst in practical applications [15], the photoactivity of the recycled nanoCoFe_2_O_4_@MC was examined toward MNZ. The nanoCoFe_2_O_4_@MC photocatalyst was first separated by an external magnet, and then was refreshed by washing with alcohol/water and dried at 100 ^○^C. The recycled photocatalyst in each run was added to a new solution of MNZ under UV-irradiation. The MNZ removal efficiency was obtained 81.59% in the first run. The results showed that the photocatalytic activity of nanoCoFe_2_O_4_@MC had an obvious reduction in the second run (78.42%) and subsequently maintained relative stability. The decreased degradation percentage may have been caused by the adsorption of intermediate products on the photocatalyst active sites, which were rendered unavailable for the degradation of a fresh MNZ solution. However, MNZ was successfully degraded at a rate of 77.58% in the fourth run of the nanoCoFe_2_O_4_@MC photocatalyst.

Moreover, the slight reduction in the MNZ degradation efficiency could be attributed to the leaching of Fe and Co metal ions and loss of Fe and Co in the photocatalytic process. For this reason, cobalt and iron ion concentrations were measured in the solution after the fourth run. However, the mentioned ions were not detected in the solution. The chemical stability of nanoCoFe_2_O_4_@MC was confirmed by the XRD analysis, showing that nanoCoFe_2_O_4_@MC did not undergo obvious changes in position, except for the intensity of diffraction peaks following the fourth run of photocatalytic recycling. This result indicated that this photocatalyst could be easily recovered.

## Concluding remarks

In summary, a new magnetic nanobiocomposite (nanoCoFe_2_O_4_@MC) was prepared as a highly potent, magnetically separable photocatalyst by a simple, fast, and new microwave-assisted method with iron and cobalt salts on MC in an alkali medium. The characterization of the magnetic nanobiocomposite confirmed pure phase spinel ferrites, spherical particle morphology with smaller agglomeration, and the ferromagnetic nature of nanoCoFe_2_O_4_@MC. The optimum conditions for the maximum MNZ removal efficiency of included pH of 11, MNZ concentration of 5 mg/L, photocatalyst loading of 0.2 g, and irradiation time of 120 min. Then, nanoCoFe_2_O_4_@MC was easily separated by a magnet and recycled without significant loss of photocatalytic activity after being used in the fourth run. The photocatalytic removal of MNZ by nanoCoFe_2_O_4_@MC is highly environmentally-friendly; has high reusability, stability, and excellent photocatalyst activity; and can be applied for the treatment of antibiotic-containing effluents.
